# TwinPE delivered as RNA or VLP enables precise deletion of GAA repeats in the *FXN* gene

**DOI:** 10.1016/j.cellin.2026.100368

**Published:** 2026-09-22

**Authors:** Xuntao Zhou, Thomas C. Todeschini, Tianxiong Yu, Runyuan Wang, Bin Liu, Annie Collins, Whitney Welch, Aditya Valji Ansodaria, Zhiping Weng, Scot A. Wolfe, Jonathan K. Watts, Erik J. Sontheimer, Wen Xue

**Affiliations:** a RNA Therapeutics Institute, University of Massachusetts Chan Medical School, Worcester, MA, USA; b Department of Genomics and Computational Biology, University of Massachusetts Chan Medical School, Worcester, MA, USA; c Department of Biological Chemistry and Pharmacology, The Ohio State University, Columbus, OH, USA; d Gene Therapy Institute, The Ohio State University, Columbus, OH, USA; e Department of Molecular, Cell and Cancer Biology, University of Massachusetts Chan Medical School, Worcester, MA, USA; f Li Weibo Institute for Rare Diseases Research, University of Massachusetts Chan Medical School, Worcester, MA, USA; g Department of Biochemistry and Molecular Biotechnology, University of Massachusetts Chan Medical School, Worcester, MA, USA; h Department of Molecular Medicine, University of Massachusetts Chan Medical School, Worcester, MA, USA

**Keywords:** Friedreich's ataxia, Neurodegenerative disorder, Prime editing, Patient-derived induced pluripotent stem cells, iPSC-derived motor neurons, Virus-like particles

## Abstract

Friedreich's ataxia (FRDA or FA) is a rare autosomal recessive neurodegenerative disorder caused by guanine-adenine-adenine (GAA) repeat expansions in the first intron of the *frataxin* (*FXN*) gene. To address this underlying genetic cause, we developed paired prime editors (twinPE) to precisely delete GAA repeats. Using a 38-bp attachment site of bacteria (attB) sequence as the reverse transcription template (RTT), we achieved a precise 1.2-kb deletion with attB insertion in HEK293T cells with a 48.0% editing efficiency. We further delivered two synthetic pegRNAs and PE6 mRNA into GM03816 FRDA fibroblasts and GM23404, as well as GM23913 patient-derived induced pluripotent stem cells (iPSCs) via electroporation, achieving average editing rates of 13.3%, 40.6%, and 25.04%, respectively. In both patient-derived iPSCs, twinPE successfully restored *FXN* expression, resulting in a ∼2-fold increase in mRNA levels, relative to non-treated controls, demonstrating a promising functional correction of gene expression. Importantly, NGS analysis revealed that >95% of all edits were precise, with minimal indels across all tested cell types. We directly delivered twinPE to iPSC-derived motor neurons (iMNs) using virus-like particles (VLPs), achieving 12.3% editing. In summary, our results demonstrate that twinPE enables efficient and precise deletion of GAA repeats across multiple cell types, including iMNs. The restoration of *FXN* mRNA in iPSCs represents a promising functional recovery, establishing a foundation for twinPE-mediated genome editing as a potential therapeutic strategy for FRDA.

## Highlights

• twinPE precisely deletes pathogenic GAA repeats in the *FXN* gene with high fidelity.
• twinPE achieves efficient editing (13.3%–40.6%) in FRDA fibroblasts and patient-derived iPSCs.
• twinPE achieves 95%–98.3% precise editing among edited reads in FRDA fibroblasts and iPSCs.
• twinPE restores *FXN* mRNA expression by approximately two-fold in patient-derived iPSCs.
• VLPs enable delivery of twinPE to post-mitotic iPSC-derived motor neurons.

## Graphical abstract

**Figure:**
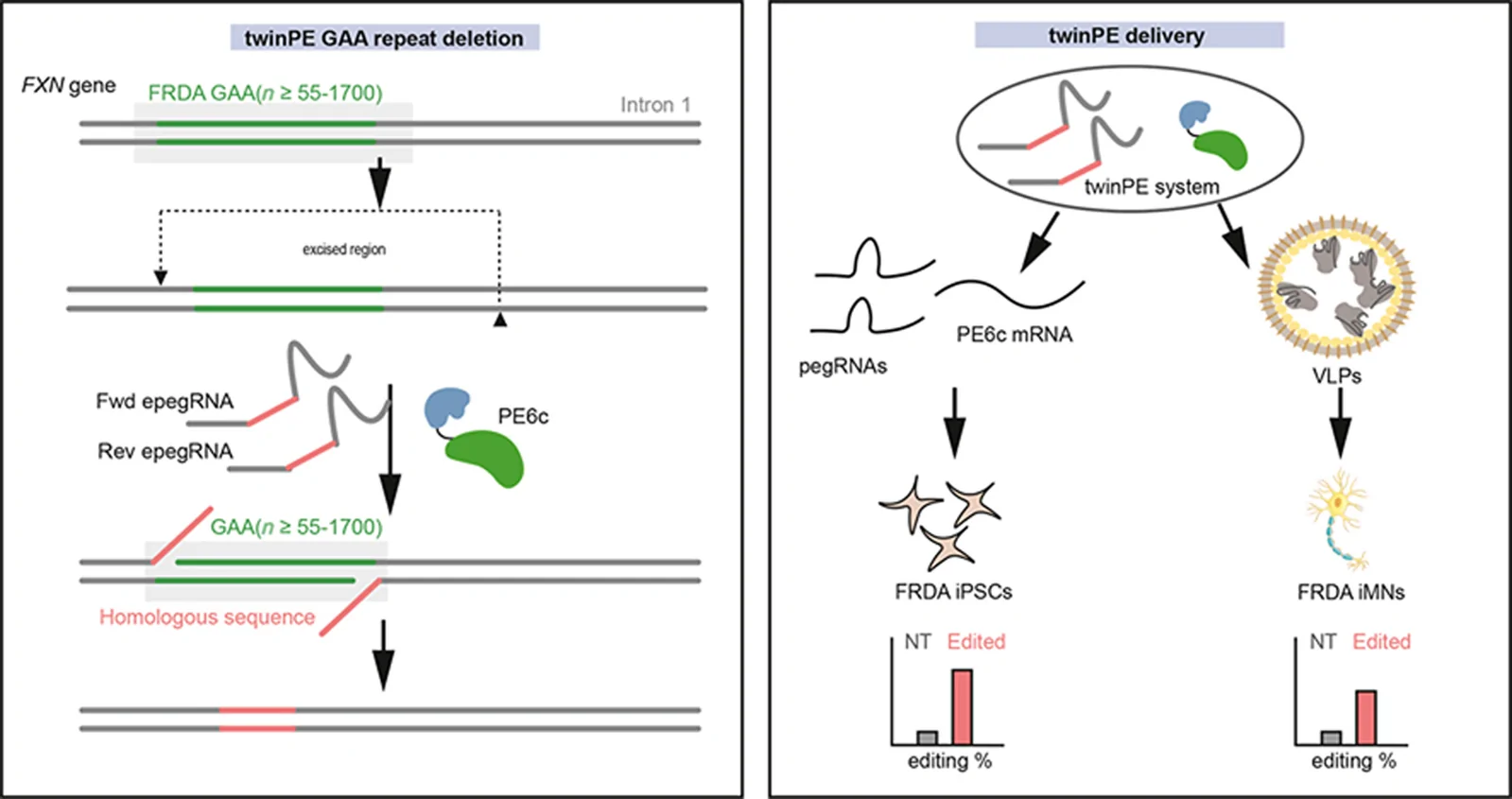

## 1 Introduction

Friedreich's ataxia (FRDA or FA) is a rare autosomal recessive neurodegenerative disorder belonging to the group of genetic disorders known as Repeat Expansion Diseases (REDs), in which repeats beyond a critical threshold lead to developmental, neurological, or neuromuscular disorders (Campuzano et al., 1996, 1997). FRDA results from expansion of a guanine-adenine-adenine (GAA) triplet repeat in the first intron of the *frataxin* (*FXN*) gene: whereas healthy individuals have 5–30 GAA repeats, FRDA patients have 55 to >1 700 repeats (Fig. 1(A)).

**Fig. 1:**
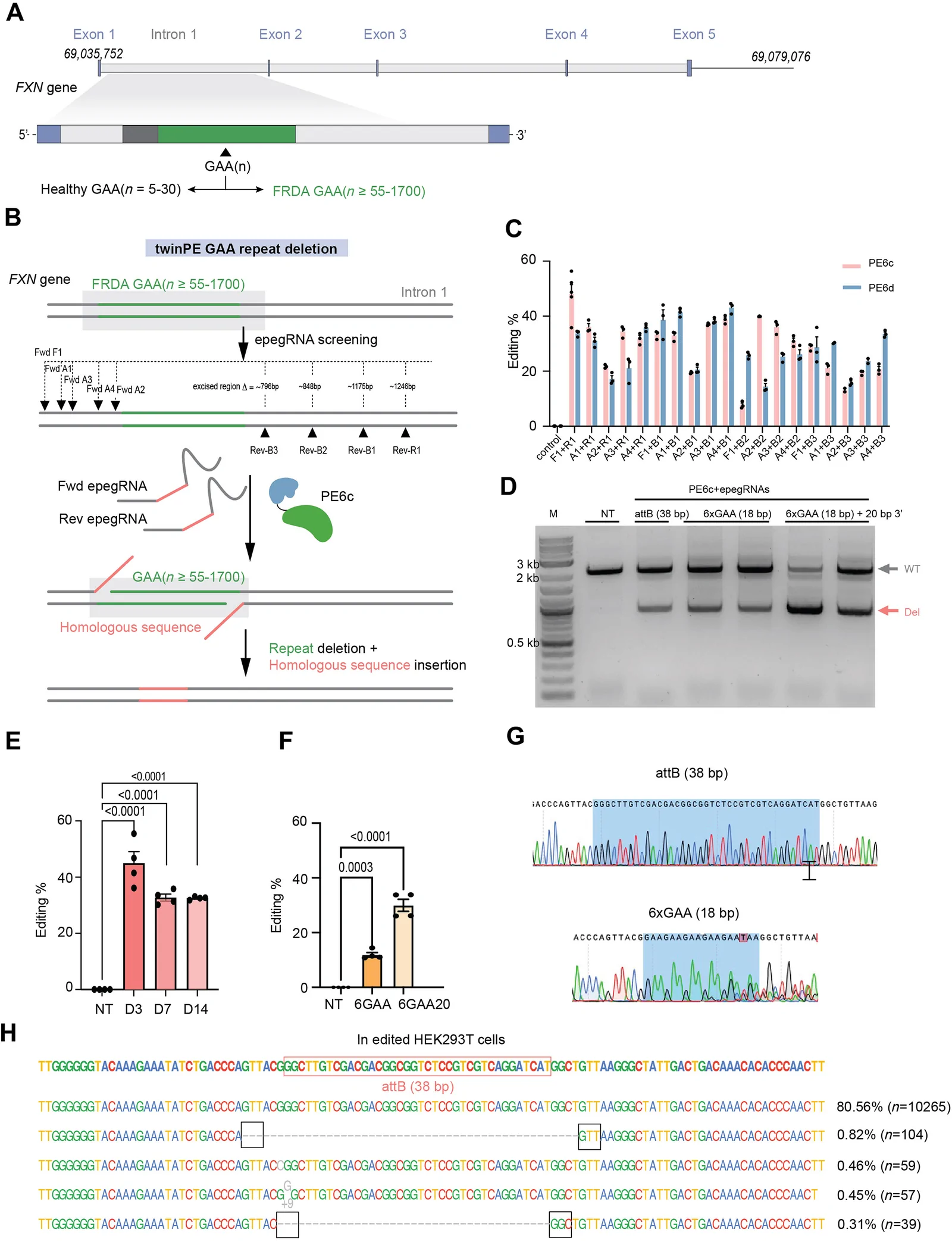
**twinPE-mediated deletion of GAA repeats in FXN intron 1.** **(A)** Schematic of the FXN gene locus, showing GAA repeats in intron 1. Healthy individuals carry 5 to 30 repeats, while FRDA patients have more than 55 repeats. **(B)** Illustration of twinPE. Two pegRNAs (fwd, forward; rev, reverse) harbor a bacterial attachment site (attB, 38-bp, salmon pink) as reverse transcriptase template (RTT), mediating a 1.2-kb deletion and insertion of attB. **(C)** Screening of epegRNA pairs. Insertion efficiency in HEK293T cells co-transfected with epegRNA pairs and either PE6c or PE6d was quantified by droplet digital polymerase chain reaction (ddPCR). An attB probe was used to detect insertion and a HEK3 locus probe was used as an internal reference control. Genomic DNA (gDNA) was extracted 3 days post-transfection (*n* = 3 biological replicates). **(D)** PCR analysis of HEK293T cells transfected with twinPE using different RTT. The wild-type (WT) band is 2097 bp. The band after deletion and insertion of attB is 888 bp. **(E)** Editing efficiency in HEK293T cells at 3, 7 and 14 days post-transfection. Data are shown as mean ± SEM (*n* = 4 biological replicates). Statistical significance was determined by an unpaired, two-tailed *t*-test with Welch's correction. **(F)** Editing efficiency with different RTT insertion in HEK293T cells at 3 days post-transfection. Data are shown as mean± SEM (*n* = 4 biological replicates). Statistical significance was determined by an unpaired, two-tailed *t*-test with Welch's correction. **(G)** Representative Sanger sequencing shows GAA repeats deletion and different RTT insertion. 6×GAA as RTT produced a more heterogenous sequence composition. **(H)** Representative deep sequencing of edited region. Black boxes indicate potential MMEJ. Deletions are indicated by dashed lines, substitutions are shown in grey.

At the molecular level, long GAA repeats can form RNA/DNA hybrids (R-loops) that impair RNA polymerase II elongation, resulting in transcriptional silencing and reduced FXN protein levels (Groh et al., 2014; Punga & Buhler, 2010; Santos et al., 2010). The resulting FXN deficiency impairs mitochondrial iron metabolism, disrupting the synthesis of iron–sulfur clusters and leading to oxidative stress. Pathological changes are most prominent in dorsal root ganglia, spinal cord, brainstem and cerebellum (Harding, 1981; Santos et al., 2010). Clinical signs include gait ataxia, muscle weakness, sensory loss, scoliosis, complex oculomotor disturbances, visual loss, hearing impairment, and hypertrophic cardiomyopathy (Harding, 1981). In advanced stages, severe locomotor impairment leads to loss of independent ambulation, and most patients require permanent wheelchair use. Furthermore, severe cardiomyopathy or arrhythmia can lead to premature mortality, with many patients not surviving beyond 40 years of age (De Michele et al., 1996; Harding, 1981).

Current therapeutic strategies, including Omaveloxolone, which activates nuclear factor erythroid 2-related factor 2 (NRF2) to protect cells from oxidative stress (Gunther et al., 2025; Lynch et al., 2019, 2023) and the AAV-based gene therapy LX2006, which increases FXN protein levels from an exogenous construct (Campuzano et al., 1996; Lynch et al., 2002), either lack specificity or carry the risk of FXN overexpression, which can drive cardiotoxicity and hepatotoxicity (Gunther et al., 2025; Huichalaf et al., 2022). Oligonucleotide-based gene activation approaches, while showing promise, have not yet proven to be sufficiently potent and specific for therapeutic use (Kilikevicius et al., 2022; Li et al., 2016; Shen et al., 2020; Wang et al., 2022). As such, there is an urgent need for therapeutic strategies that delete the GAA repeat expansion and thus directly target the underlying genetic cause of FRDA within its endogenous regulatory context.

Previous studies have reported deletion or interruption of GAA repeats using Cas9 nucleases or base editor (Matuszek et al., 2025; Mishra et al., 2024; Rocca et al., 2020). For example, Cherqui et al. reported that Cas9-mediated deletion of repeats in FRDA patient iPSCs can prevent the FRDA-associated mitochondrial phenotype in iPSC-derived neurons (Mishra et al., 2024). However, many Cas9 approaches are limited by increased off-target edits, unintended indels and potentially toxic double-strand break (DSBs) (Mazzara et al., 2020). By contrast, Prime Editor—a Cas9 nickase paired with an engineered Moloney murine leukemia virus reverse transcriptase (MMLV) RT and a PE guide RNA (pegRNA), can install diverse substitutions, small insertions, or deletions without inducing DSBs (Anzalone et al., 2019). Meanwhile, we and others have developed PEDAR, PRIME-Del, or twinPE in which a pair of pegRNAs directs precise genomic deletion (Anzalone et al., 2022; Choi et al., 2022; Jiang et al., 2022). More recently, Doman et al. found pairing Cas9 nickase with an evolved RT from the Tf1 retrotransposon (PE6c) enhances editing at a variety of targets compared to evolved PE variants that use MMLV RT (*e.g*., PE2, PE3, and PEmax) (Doman et al., 2023).

In this study, we show that twinPE, using PE6c, can be used to precisely delete GAA repeats across different cell types and restore FXN protein expression. Moreover, we employ therapeutically relevant virus-like particles (VLPs) to deliver twinPE for GAA repeat deletion in patient-derived induced pluripotent stem cells (iPSCs) and iPSC-derived neurons, reflecting the key neurological contribution to the pathology of FRDA.

## 2 Results

To screen for optimal spacer sequences to delete the GAA repeat tract in *FXN* intron 1, we designed five forward and four reverse spacers flanking the GAA repeat region (Fig. 1(B)). We first tested 38-bp attachment site of bacteria (attB) as a reverse transcription template (RTT) because the attB sequence has been shown to induce efficient deletion by twinPE and attB insertion within introns would be unlikely to affect gene expression (Anzalone et al., 2022). We tested a panel of 19 engineered pegRNA (epegRNA) pairs with PE6c or PE6d effector in HEK293T cells and quantified deletion efficiency by droplet digital PCR (ddPCR). The pegRNAs F1 and R1 showed the highest deletion efficiency, with approximately 48.0% editing in HEK293T cells with PE6c (Fig. 1(C) and Supplementary Fig. 1a–b). Measuring editing efficiency on days 3, 7, and 14 post-transfection suggested that repeat tract deletions were stable (Fig. 1(E)). To evaluate whether editing efficiency would be enhanced by the RTT composed of either 6×GAA repeats (18 bp) or 6×GAA repeats with a 20-bp 3′ end (homology flap matching the downstream genomic sequences), we transfected HEK293T cells with the corresponding epegRNA plasmids and PE6c plasmid. We observed that 6×GAA repeats (18 bp) or 6×GAA repeats with an additional 20-bp at the 3′ end also induced deletion bands, and their editing efficiencies were still lower than the attB RTT (Fig. 1(D–F)). Additionally, Sanger sequencing showed more heterogenous sequence composition compared to attB (Fig. 1(G)). We hypothesized that using a repetitive sequence as the RTT might cause “slippery” hybridization between repeats, resulting in variable sequences in the edited cell population. We therefore chose attB as the RTT for follow-up studies. In deep sequencing (NGS) analyses, most reads consisted of a deletion plus attB insertion (Fig. 1(H)). However, some reads lacked the full insertion and instead exhibit deletions between the targeted genomic sequences joined by microhomology (e.g., “GTT” or “GGC”), suggesting the involvement of the microhomology-mediated end joining (MMEJ) repair pathway (Truong et al., 2013) (Fig. 1(H)).

To examine potential off-target (OT) sites for each pegRNA, we employed the *in silico* prediction method Cas-OFFinder (Bae et al., 2014). We selected all potential OT sites containing ≤1-nt DNA or RNA bulges and ≤3-nt mismatches (Bravo et al., 2022) (Supplementary Fig. 2a). Junction PCR analysis at each OT site in non-treated cells and twinPE-edited cells did not reveal any expected junction band (Supplementary Fig. 2b–d), nor did NGS analysis identify attB insertion at any OT site (Supplementary Fig. 2e–f).

GM03816 FRDA patient-derived fibroblasts harbor around 330 GAA repeats in one *FXN* allele and 380 repeats in the other, providing a genetic system to test whether twinPE can mediate deletion of pathogenic GAA repeats. We developed an all RNA-based twinPE system, preparing PE6c mRNA by *in vitro* transcription (IVT) with N1-methylpseudouridine and CleanCap AG (3′-OMe) and synthesizing F1 and R1 pegRNAs with three 2′-O-methyl and 3′-phosphorothioate modifications at each end to enhance stability (Fig. 2(A)). The RNA components were delivered into HEK293T cells or GM03816 fibroblasts via electroporation. PCR analyses confirmed the expected deletion (Fig. 2(B–C)). Electroporation of Cas9 mRNA and two sgRNAs bearing the same spacer sequences as the F1 and R1 pegRNAs yielded a shorter PCR product, corresponding to the deletion without the attB insertion (Fig. 2(B–C) and Supplementary Fig. 3a-b). To determine whether editing efficiency in fibroblasts depends on the amount of PE6c effector, we performed a dose-response experiment using 0.5, 1.0, or 1.3 μg of PE6c mRNA, while maintaining a fixed ratio of effector and pegRNAs, as in the previous study (Liu et al., 2022). ddPCR analysis indicated that editing efficiency reached a 13.3% at 1.3 μg of PE6c mRNA (Fig. 2(D)). In edited FRDA fibroblasts, NGS analysis showed most reads contained a deletion with attB insertion (Fig. 2(E)). TwinPE is thus compatible with an all-RNA delivery format by electroporation into patient-derived fibroblasts.

**Fig. 2:**
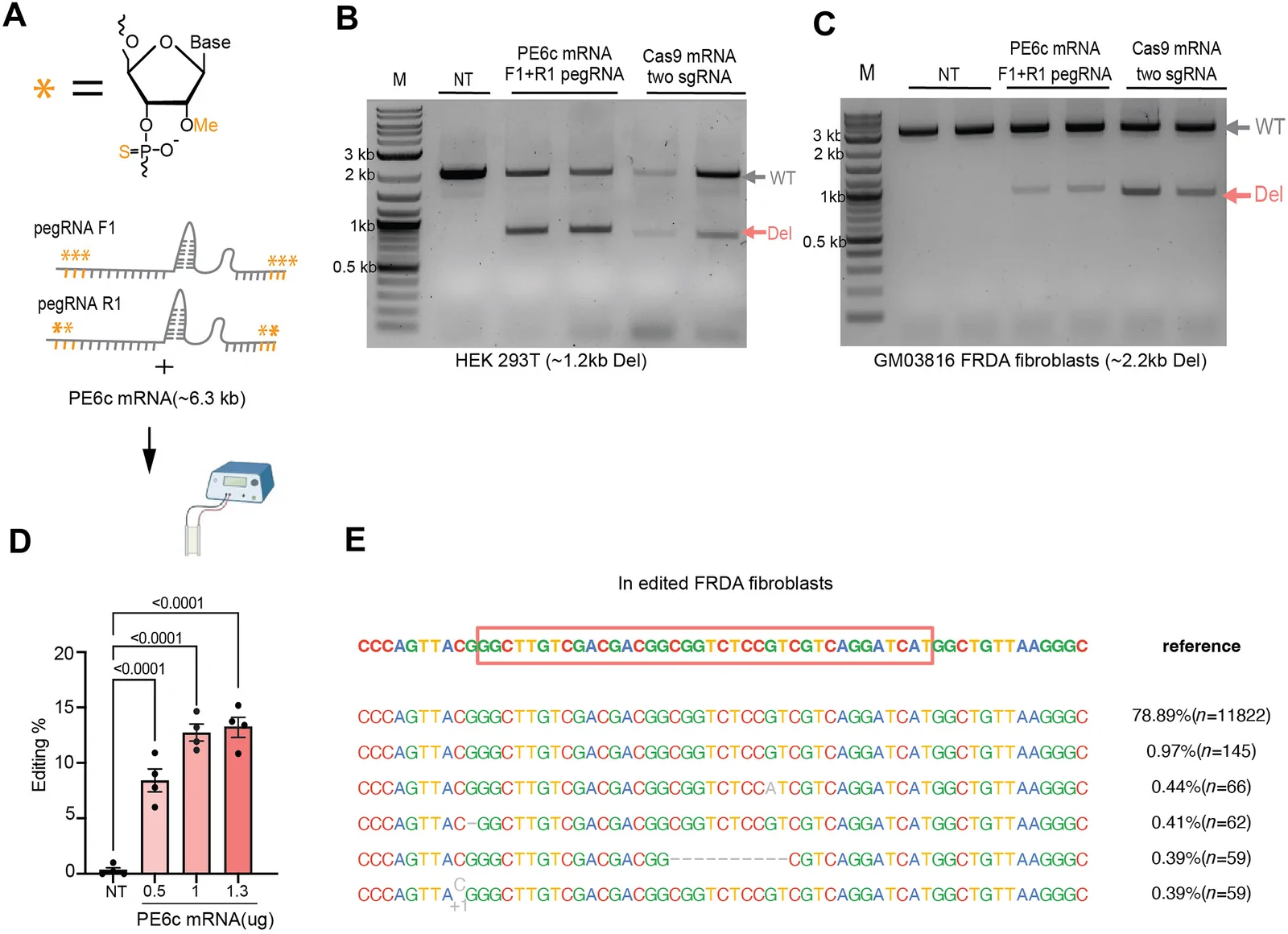
**twinPE-mediated GAA repeat deletion in GM03816 patient-derived fibroblasts.** **(A)** Schematic of twinPE-mediated deletion of GAA repeats in FRDA fibroblasts. GM03816 fibroblasts (with 330/380 GAA repeats) were co-electroporated with in vitro-transcribed (IVT) PE6c mRNA and 2′-O-methyl, 3′-phosphorothioate-modified pegRNAs, resulting in a 2.2-kb deletion and insertion of attB. **(B,C)** PCR analysis of HEK293T cells (B) or fibroblasts (C) electroporated with PE6c mRNA plus pegRNA pairs or Cas9 mRNA plus sgRNA pairs. The band after deletion and insertion of attB is 888 bp. The band after deletion only (Cas9 mRNA with sgRNA pairs) is 850 bp. **(D)** FRDA fibroblasts were electroporated with a fixed ratio of PE6c mRNA (*e.g*., 1 000 ng) to pegRNA (*e.g*., 100 pmol each for pegRNA F1 and R1). Editing efficiency was quantified by ddPCR. Data are shown as mean ± SEM (*n* = 4 biological replicates). Statistical significance was determined by an unpaired, two-tailed *t*-test with Welch's correction. **(E)** Representative deep sequencing of edited region in FRDA fibroblasts. Deletions are indicated by dashed lines, substitutions are shown in grey. The attB sequence is highlighted in pink box.

Compared to patient fibroblasts, fibroblast-derived induced pluripotent stem cells (iPSCs) have greater therapeutic potential due to their ability to differentiate into many cell types (e.g., neurons). To evaluate whether twinPE could delete GAA repeats in iPSCs, we electroporated GM23404 [hereafter iPSC (330/380)] with F1 and R1 pegRNAs and PE6c mRNA. PCR analyses confirmed the 888-bp edited product and the ∼3-kb wild-type product in iPSC (330/380) (Fig. 3(A)). The editing efficiency in iPSC (330/380) was approximately 40.6 % ± 1.8% at 3-days post-electroporation (*n* = 4, Fig. 3(B)) and 43.5 % ± 4.3% at 14 days post-electroporation (*n* = 4, Fig. 3(B)), suggesting stability of the repeat tract deletion. We also noticed substantial cell death and severely reduced cell proliferation after electroporation when the amount of Cas9 mRNA was kept the same as that of PE6c mRNA. Thus, we used a lower amount of Cas9 mRNA to maintain a reasonable proliferation rate of iPSCs for ddPCR and RT-qPCR analyses (Fig. 3(C) and Supplementary Fig. 3c). RT-qPCR analysis showed that FXN mRNA expression increased approximately two-fold in twinPE-treated cells compared to untreated cells (Fig. 3(C)). By contrast, FXN mRNA expression did not increase in Cas9-treated cells (Fig. 3(C)).

**Fig. 3:**
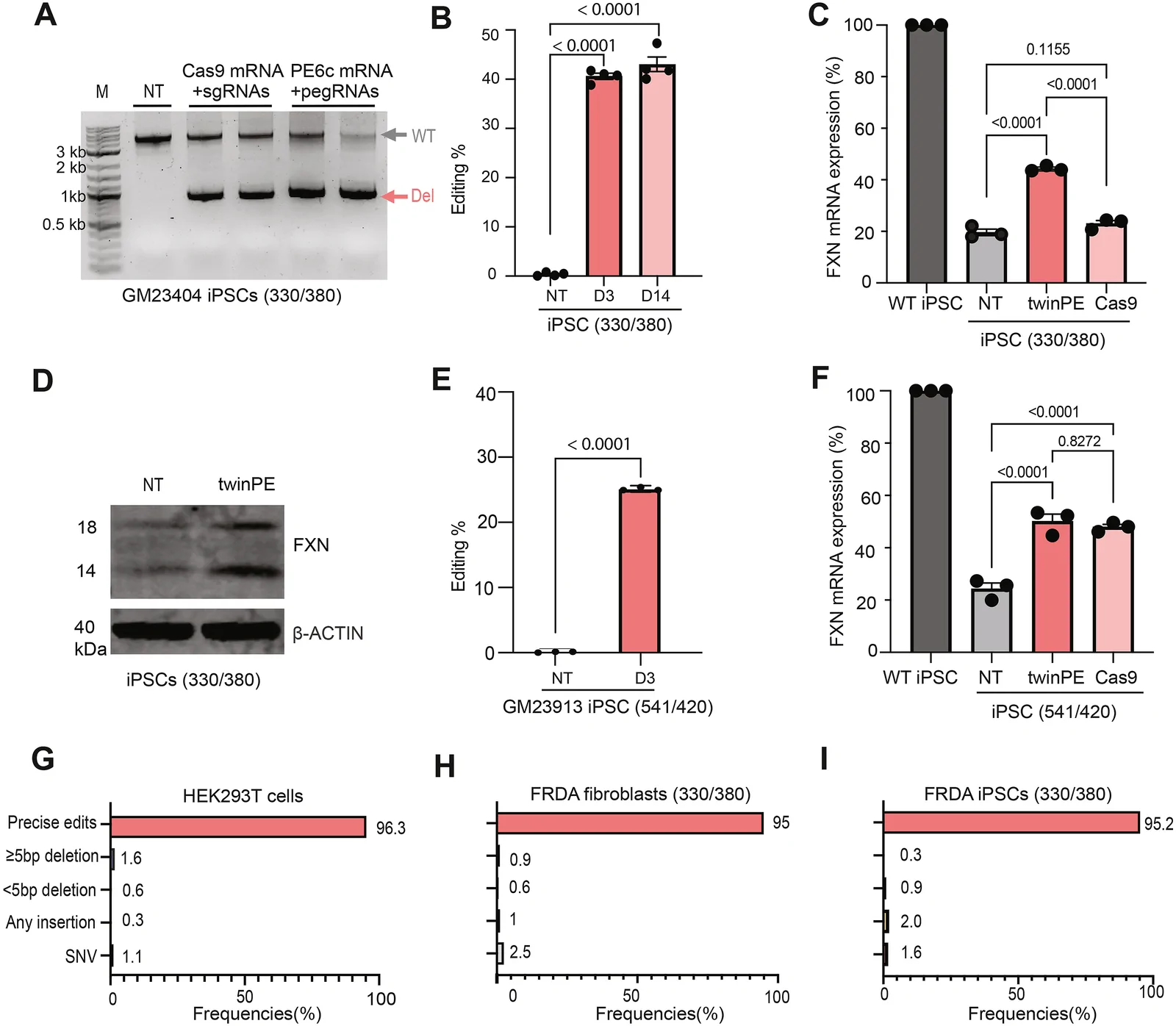
**twinPE-mediated GAA repeat deletion in GM23404 iPSCs.** **(A)** PCR analysis of GM23404 iPSCs electroporated with PE6c mRNA plus pegRNA pairs or Cas9 mRNA plus sgRNA pairs. The band after deletion and insertion of attB is 888 bp. The band only after deletion (Cas9 mRNA) is 850 bp. **(B)** Editing efficiency at days 3 and 14 post-transfection was measured by ddPCR. Data are presented as mean ± SEM (*n* = 4 biological replicates). NT, non-treated. Statistical significance was determined by an unpaired, two-tailed *t*-test with Welch's correction. **(C)***FXN* mRNA expression in FRDA iPSCs (330/380) after editing (using twinPE or Cas9), measured 3 days after electroporation and normalized to Actin mRNA. Data are presented as mean ± SEM (*n* = 3 biological triplicates). WT iPSC, GM25256 healthy WT iPSCs; NT, non-treated iPSCs. **(D)** Representative Western blot demonstrating restoration of frataxin protein (18 kDa and 14 kDa) in FRDA iPSCs. β-Actin protein was a loading control. **(E)** Editing efficiency at day 3 post-transfection was measured by ddPCR. Data are presented as mean ± SEM (*n* = 3 biological replicates). **(F)** FXN mRNA expression in FRDA iPSCs (541/420) (twinPE or Cas9) measured 3 days after electroporation and normalized to Actin mRNA. Data are presented as mean ± SEM (*n* = 3 biological triplicates). **(G–I)** Distribution of edited allele subtypes by deep sequencing data of 187 bp PCR amplicons at the FXN locus in **(G)** HEK293T cells, **(H)** FRDA fibroblasts, and **(I)** GM23404 iPSCs. Using a custom analysis method, sequencing reads were categorized based on redefined criteria for detecting sequence changes in the edited region (attB 38 bp ± 5 bp). The defined subtypes are: ≥5 bp deletion, deletions of 5 bp or more; <5 bp deletion, short deletions of 1–4 bp; Any insertion, Insertions of any length; SNV, single nucleotide variants. Note that these subtypes are not mutually exclusive; a single read can be counted in more than one category (e.g., a read with both an insertion and an SNV).

Western blot analysis revealed that twinPE increased expression of the intermediate frataxin (FXN 42–210, 18 kDa) from 56.3% in non-treated iPSCs to 93.2%, and increased expression of the mature frataxin isoform (FXN 81–210, 14 kDa) from 36.7% to 66.0% (Fig. 3(D) and Supplementary Fig. 3d), indicative of partially restored protein expression following deletion of GAA repeats in the iPSC population.

To extend our work to additional cell lines and repeat lengths, we tested a second iPSC line containing alleles with around 541 and 420 repeats, GM23913 [hereafter iPSC (420/541)]. We treated these cells with F1 and R1 pegRNAs along with PE6c mRNA. We observed that this twinPE mediated 25.04% deletion (Fig. 3(E)). This is slightly lower than was observed for the iPSC (330/380) cells, though still of therapeutic relevance even before optimization of delivery or other parameters for this cell line. We also observed that *FXN* mRNA expression increased from 24.3% to 50.1% after editing in this iPSC line (Fig. 3(F)).

Thus, twinPE-mediated deletion of GAA repeats can restore FXN expression at both the mRNA and protein levels in edited FRDA iPSCs. These results are therapeutically promising given that restoring as little as 20% of healthy gene expression is considered likely to be of therapeutic value (Payne, 2022) and that cell-autonomous effects drive therapeutic benefit even if expression is restored in as few as 50% of cardiomyocytes (Belbellaa et al., 2019). To determine the precision of genomic deletions, we performed NGS analysis. All reads were examined for single nucleotide variants, deletions, and random insertions around the edited regions (attB ± 5 bp). The analysis showed that over 95% of edited reads across HEK293T cells, FRDA fibroblasts, and FRDA iPSCs correspond to perfect intended edits (Fig. 3(G–I) and Supplementary Fig. 4a-b).

For clinical applications, iPSCs are of limited relevance and we require editing to be effective in neurons. To determine whether twinPE could directly edit iPSC-derived motor neurons (iMNs), we differentiated FRDA iPSCs (330/380) into FRDA iMNs (Fig. 4(A)) and assessed common transfection methods, including MessengerMAX and electroporation. Electroporation resulted in substantial cell death and achieved only 1%–2% editing efficiency (Fig. 4(B)). Although MessengerMAX exhibited lower toxicity, editing efficiency remained modest at approximately 5% (Fig. 4(B)).

**Fig. 4:**
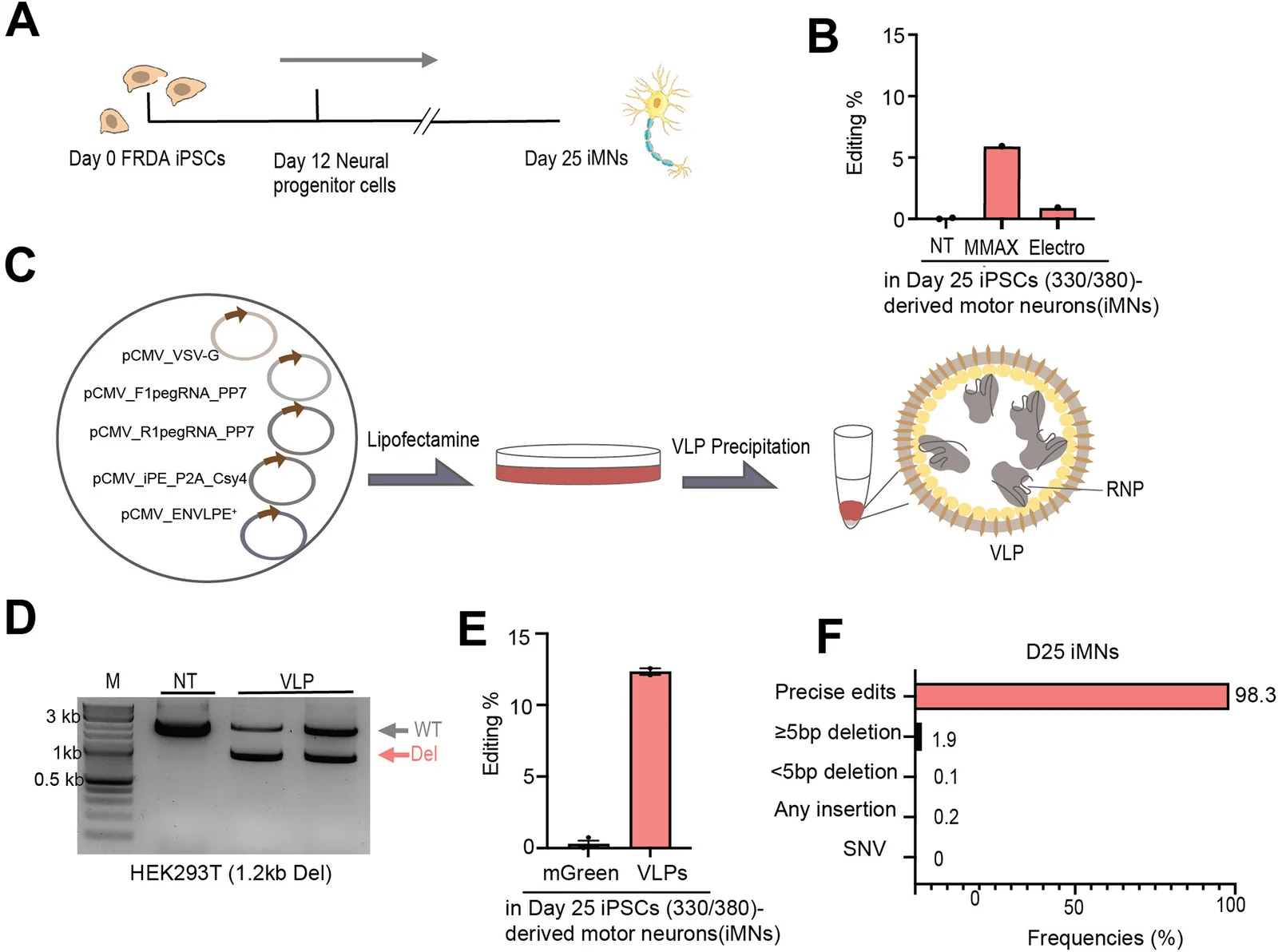
**Delivery of twinPE to FRDA iPSC-derived motor neurons (iMNs) using virus-like particles (VLPs).** **(A)** Schematic representing the differentiation of iPSCs into iMNs. **(B)** Deletion efficiency in iMNs was quantified by ddPCR (MMAX: MessengerMAX transfection, *n* = 1; Electro: Electroporation, *n* = 1). **(C)** Schematic of production of VLPs (VLPs: ENVLPE^+^). HEK293FS cells were transfected with plasmids encoding VSV-G, Gag-Pol, iPE-P2A-Csy4, and two pegRNAs. After 36 h, supernatant was collected and centrifuged to precipitate VLPs. **(D)** PCR from HEK293T cells transduced with ENVLPE + packaged PE, F1 and R1 pegRNA. The band after deletion and insertion of attB is 888 bp. **(E)** Deletion efficiency in iMNs was quantified by ddPCR (mGreen: ENVLPE + packages mGreenLantern; VLP: ENVLPE + packages pegRNA pairs and PE). Data are presented as mean ± SEM (*n* = 3 biological replicates for mGreen; *n* = 2 biological replicates for VLP). **(F)** Distribution of edited *FXN* allele subtypes in iMNs was determined by deep sequencing in PCR amplicons (187 bp) at FXN locus within the edited region (attB ± 5 bp) and the flanking regions (positions 5–40 and 97–187), ≥5 bp deletion, deletions of 5 bp or more; <5 bp deletion, short deletions of 1–4 bp; Any insertion, insertions of any length; SNV, single nucleotide variants.

As an alternative delivery strategy, we tested VLPs, which can package gene-editing payloads as ribonucleoprotein (RNP) complexes lacking viral genetic material. We compared two VLP systems: Friend murine leukemia virus (FMLV) VLPs and engineered nucleocytosolic vesicles (ENVLPE^+^) VLPs (Geilenkeuser et al., 2025; Ramadoss et al., 2025). To generate TwinPE–loaded ENVLPE^+^ VLPs, we transfected producer cells with plasmids encoding pCMV_VSV-G, pCMV_F1_pegRNA_PP7, pCMV_R1_pegRNA_PP7, pCMV_iPE_P2A_Csy4, and pCMV_ENVLPE^+^. The supernatant was then collected and concentrated using PEG-it solution (Fig. 4(C)). We first tested the ability of VLPs to deliver twinPE to HEK293T cells; PCR analysis confirmed the expected deletion in HEK293T cells transduced with different VLP types, such as FMLV VLP (Fig. 4(D) and Supplementary Fig. 5a-b). ddPCR analysis showed that ENVLPE^+^ VLPs outperformed FMLV VLPs in FRDA iPSCs (Supplementary Fig. 5c). Indeed, four days after transduction of iMNs with ENVLPE^+^ VLPs carrying mGreenLantern, we observed GFP-positive iMNs, demonstrating that the VLPs could successfully deliver cargo into neurons (Supplementary Fig. 6). Importantly, ddPCR analysis in iMNs transduced with ENVLPE^+^ VLPs carrying twinPE revealed an editing efficiency of 12.3%, and NGS data demonstrated that over 98% of edited reads correspond to perfect intended edits (Fig. 4(E–F)).

## 3 Discussion

In this study, we demonstrated the therapeutic potential of twinPE by achieving precise deletion of GAA repeats in HEK293T cells, FRDA fibroblasts, patient-derived iPSCs, and iPSC-derived iMNs. Our pegRNAs were designed to target only intronic regions, thereby avoiding perturbation of *FXN* coding sequences. We achieved editing efficiencies of 48.0% in HEK293T cells, 13.3% in fibroblasts, 40.6% in iPSC (330/380), 25.04% in iPSC (420/541) and 12.3% in iMNs. Notably, this approach demonstrates twinPE editing efficacy in a post-mitotic cell model (*e.g*., iMNs)—a critical requirement for most *in vivo* applications. Importantly, the proportion of precise editing was consistently high across all cell types (95%–98.3%), underscoring the superior fidelity of twinPE over DSB-dependent nuclease editors. In addition, NGS analysis suggested the potential involvement of the microhomology-mediated end joining (MMEJ) in HEK293T cells, providing mechanistic insight into off-pathway editing outcomes. Given recent evidence that MMEJ inhibition combined with a DNA-PK inhibitor can improve the efficiency of precise genome knock-in, future work could explore whether treatment with MMEJ inhibitors or dual MMEJ/DNA-PK inhibitors can modulate the precision and efficiency of GAA repeat contraction and attB integration (Liu et al., 2026; Wimberger et al., 2023).

We demonstrated that the attB sequence is an effective RTT to induce genomic deletions in the context of twinPE compared with 6×GAA repeat sequence. We also observed that attB significantly reduces noisy deletions caused by “slippery” hybridization, e.g., when using 6×GAA repeats or 6×GAA with a 20-nt 3′ overhang as RTT. Notably, these RTT variants differ substantially in GC content—63% for attB, 33% for 6×GAA, and 37% for 6×GAA with a 20-nt 3′ overhang—suggesting that GC composition might affect editing fidelity and efficiency. Therefore, a future investigation could explore how additional RTT sequences (e.g., varying in GC content) affect twinPE outcomes. We also found that F1 and R1 spacers were the most effective pegRNA pair in our screening, despite being the most widely spaced among the tested pegRNA pairs (Fig. 1(B–C)). Mazzara et al. previously demonstrated that using Cas9 nuclease to delete the entire intron 1, which contains the expanded GAA repeat sequence, resulted in a better rescue effect than removal of the pathogenic GAA repeat tract. This finding suggests that complete removal of the GAA repeats does not adversely affect FXN expression. Further, removal of FXN intron 1 could eliminate additional repressive chromatin, thereby facilitating reactivation of the FXN gene (Mazzara et al., 2020).

A previous study used plasmid DNA electroporation to deliver paired prime editors into iPSC models carrying repeat expansions in the *AR*, *SCA1* and *ATXN1* genes (Hwang et al., 2024). Our work establishes that an all-RNA delivery approach is feasible in FRDA iPSC models. In addition, we demonstrated that virus-like particles (VLPs) can successfully deliver the twinPE system directly into iMNs, achieving an editing efficiency significantly higher than that achieved with lipid-mediated transfection (MessengerMAX) or electroporation. To our knowledge, this work is the first demonstration of PE6c-mediated GAA repeat deletion in FRDA patient-derived iPSCs and iMNs via VLP delivery. Although VLPs achieved higher editing efficiency than MessengerMAX or electroporation, the rate was still lower than that achieved in iPSCs. This is likely due to the difficulty of transducing iMNs, as dense perineuronal nets (PNNs) surrounding neurons can impede viral access to the neuronal cell surface (Wanisch et al., 2013). CRISPR-Cas9 amphiphilic nanocomplexes have been used to target *Bace1* as a therapeutic strategy for Alzheimer's disease, achieving around 30%–40% deletion efficiency in mouse primary neurons (Park et al., 2019). Compared to nanocomplexes, VLPs remain a promising delivery tool for neurons because neurons express low-density lipoprotein receptor (LDLR) and can be efficiently transduced by VSV-G-pseudotyped VLPs (Hastie et al., 2013). Further optimization of VLP design—for instance, through the incorporation of cell-penetrating peptides or neuron-specific targeting antibodies or proteins—could enhance transduction efficiency and reduce immune response. YG8-800 is a promising mouse model for testing our strategy *in vivo.* Tremblay et al. demonstrated that these mice showed a progressive neuromuscular degeneration and heart hypertrophy (Gerard et al., 2023). Thus, future studies will explore delivering twinPE into FRDA animal models with possible delivery strategies, including dual-AAV, LNP, and VLP.

In summary, our findings establish twinPE, delivered as either RNA or VLPs, as a versatile and effective strategy for GAA repeat contraction to restore FXN expression. This study establishes a foundation for twinPE-mediated genome editing as a potential therapeutic strategy for FRDA and other repeat expansion diseases.

## 4 Materials and methods

### 4.1 Cell culture and neuron differentiation

HEK293T cells were obtained from ATCC and cultured at 37 °C with 5% CO_2_ in DMEM medium (Gibco) supplemented with 10% FBS and 1% Penicillin-Streptomycin (5 000 U/mL, Gibco).

GM03816 fibroblasts (carrying alleles with 330 and 380 GAA repeats), GM23404 FRDA iPSC (derived from GM03816 fibroblasts), and GM23913 (carrying alleles of approximately 541 and 420 repeats) were purchased from the Coriell Institute for Medical Research. GM03816 was cultured at 37 °C with 5% CO_2_ in Eagle's Minimum Essential Medium with Earle's salts (ATCC, Lot # 30-2003), 1% non-essential amino acids (Gibco), 10% FBS, and 1% 5 000 U/mL Penicillin-Streptomycin (Gibco). GM23404 was cultured at 37 °C with 5% CO_2_ in mTeSR Plus medium and was plated on Matrigel-coated plates, and the medium was changed every 2 days. Differentiation of iPSCs into motor neurons (iMNs) was performed according to a previously established protocol (Baskerville et al., 2024). 250 mL Stage 1 (S1) media: 118.75 mL IMDM (Life Technologies, 12440053), 118.75 mL F12 (Life Technologies, 11765062), 2.5 mL NEAA (Life Technologies, 11140050), 5 mL B27 (Invitrogen, 17504044), 2.5 mL N2 (Life Technologies, 17502048), 2.5 mL PSA (Invitrogen, 15140122), 25 μL LDN193189 (Stemgent, 04007402), 250 μL SB431542 (STEMCELL Technologies, 72234), 50 μL CHIR99021 (Sigma-Aldrich, SML1046). 250mL Stage 2 (S2) media: 118.75 mL IMDM (Life Technologies, 12440053), 118.75 mL F12 (Life Technologies, 11765062), 2.5 mL NEAA (Life Technologies, 11140050), 5 mL B27 (Invitrogen, 17504044), 2.5 mL N2 (Life Technologies, 17502048), 2.5 mL PSA (Invitrogen, 15140122), 25 μL LDN193189 (Stemgent, 04007402), 250 μL SB431542 (STEMCELL Technologies 72234), 50 μL CHIR99021(Sigma-Aldrich SML1046), 2.5 μL All-trans RA (Sigma-Aldrich R2625), 2.5 μL SAG (Cayman Chemicals 11914). 250 mL Stage 3 (S3) media: 118.75mL IMDM (Life Technologies 12440053), 118.75mL F12 (Life Technologies 11765062), 2.5mL NEAA (Life Technologies 11140050), 5mL B27 (Invitrogen 17504044), 2.5mL N2 (Life Technologies 17502048), 2.5mL PSA (Invitrogen 15140122), 25 μL Compound E (Millipore 565790), 31.3 μL DAPT (Sigma Aldrich D5942), 2.5 μL db-cAMP (Millipore 28745), 12.5 μL All-trans RA (Sigma Aldrich R2625), 0.25 μL SAG (Cayman Chemicals 11914), 100 μL Ascorbic Acid (Sigma Aldrich A4544), 250 μL BDNF (PeproTech 45002), 250 μL GDNF (PeproTech 45010). FRDA iPSCs were seeded in Matrigel-coated 6-well plates with 1 mL mTeSR Plus medium. When the cells were at 80% confluence, 5 × 10^5^ iPSCs were seeded in Matrigel-coated 6-well plates with 2 mL pre-warmed S1 medium and 4 μL 10 mM ROCK inhibitor (Y) on Day 0. On Day 1, S1 + Y medium was removed, and S1 medium was added to each well. S1 medium was exchanged again on Days 3 and 5 (2.5 mL per well). On Day 6, S1 medium was removed, and 1 mL Accutase was added to each well, followed by washing with PBS. 2 × 10^6^ cells were resuspended in 5 mL S2 medium with 10 μL ROCK inhibitor (Y) and seeded into a Matrigel-coated T25 flask. On Day 7, S2 + Y medium was removed, and S2 medium was added to the T25 flask. S2 medium was changed again on Days 9 and 11. On Day 12, 5 × 10^6^ cells were seeded in 2.5 mL S3 medium with 5 μL ROCK inhibitor (Y) in each well of a 6-well plate. On Day 13, S3 + Y medium was replaced with S3 medium alone. S3 medium was changed twice per week (e.g., Monday/Thursday or Tuesday/Friday) until completion of the experiment.

### 4.2 Plasmids

PE6c (Addgene #207853) and PE6d (Addgene #207854) plasmids were gifts from Dr. David Liu. pCMV_ENVLPE^+^ (Addgene #232427), pCMV_iPE-C_P2A_Csy4 (Addgene #232428), and pCMV_mGL_PP7 (Addgene #232440) plasmids were gifts from Dr. Dong-Jiunn Jeffery Truong.

epegRNA expression plasmids were constructed using a custom vector (BfuAI- and EcoRV-digested) that has been described in previous studies. The gBlocks gene fragments with spacer, guide scaffold, RTT and PBS sequence were synthesized by Integrated DNA Technologies (IDT), followed by Gibson assembly using Gibson Assembly Master Mix (New England Biolabs). Colonies were selected and further confirmed by Sanger sequencing using the human U6, M13R, and other specific primers. Sequences of epegRNAs are listed in Supplementary Table 1.

The pU6_FXN_F1 and pU6_FXN_R1 pegRNA VLP plasmids were constructed using the pU6_HEK3+1T > A_(PP7-C4-Q1) vector (Addgene #232437) as a backbone. The FXN F1 or R1 pegRNA gene fragments from IDT were inserted into the linearized vector via Gibson assembly.

### 4.3 Transfection and electroporation

Cells were seeded in 12-well plates. 24 h post-seeding, cells were transfected using Lipofectamine 3000 according to the manufacturer's protocol, with a mixture of 1 000 ng PE6c plasmid and 500 ng of each epegRNA plasmid. Genomic DNA (gDNA) was harvested from each well using 100 μL of QuickExtract DNA Extraction Solution (Sigma, #QE09050) on day 3 post-transfection.

For pegRNA screening, cells were seeded in 96-well plates and transfected with 100 ng of PE6c or PE6d plasmid using Lipofectamine 2000 according to the manufacturer's instructions. gDNA was collected on day 3 by adding 50 μL of QuickExtract solution per well.

For Neon electroporation, a mixture of 5 000 cells, 1 000 ng of PE6c mRNA, and 100 pmol of each pegRNA in Buffer R was electroporated using a 10 μL Neon tip. We note that to avoid cytotoxicity, we needed to use four-fold less Cas9 mRNA compared to PE6c mRNA; the amount of guide RNA was kept the same. We used 250 ng of Cas9 mRNA and 50 pmol of each sgRNAs for electroporation in iPSCs for ddPCR and RT-qPCR analyses.

### 4.4 Genomic DNA isolation

72 h post-transfection or electroporation, gDNA in Quick Extract solution was incubated in a thermocycler at 65 °C for 15 min, followed by 98 °C for 5 min, as per the manufacturer's protocol.

### 4.5 Electrophoresis and Sanger sequencing

PCR amplification was performed at the *FXN* locus using TaKaRa LA Taq® DNA Polymerase Hot-Start Version (TakaraBio, Cat. No. RR042A) and *FXN* PCR primers (Forward primer 5′ – TGGTTCTCCCGGTTGCATTT-3′; Reverse primer 5′ – AGGGGGAGCTTAGGGTCAAT-3′).

PCR was performed according to the manufacturer's manual under the following conditions: 94 °C for 1 min, 30 cycles of 98 °C for 10 s and 68 °C for 3 min, followed by 72 °C for 10 min. PCR products were purified using agarose gel electrophoresis.

### 4.6 Real-time quantitative PCR (RT-qPCR)

Total RNA was extracted using the PureLink™ RNA Mini Kit (Thermo Fisher Scientific, #12183018A). cDNA was synthesized from the RNA using MultiScribe™ Reverse Transcriptase (Invitrogen, #4311235) using random primers (Invitrogen, # 48190011), according to the manufacturer's protocol.

### 4.7 Reverse transcription

25 °C for 10 min; 37 °C for 120 min; 85 °C for 5 min

qPCR primers are listed in Supplementary Table 2 qPCR reactions were performed using SsoFast™ EvaGreen® Supermix (Bio-Rad, #1725200) according to the manufacturer's instructions.

qPCR:

98 °C for 120 sec
98 °C for 5 sec; 60 °C for 5sec (30× cycles)
65 °C–95 °C for 2–5 sec/step

### 4.8 *In vitro* transcription

PE6c mRNA was transcribed *in vitro* from a linearized IVT PE6c plasmid using the HiScribe™ T7 High Yield RNA Synthesis Kit (NEB, #E2040S) with N1-MethylpseudoUTP (TriLink, #N-1081-10) and CleanCap AG (3′-OME) (TriLink, #N-7113-10).

### 4.9 Droplet digital PCR (ddPCR)

The efficiency of precise attB insertion was quantified by ddPCR relative to a reference amplicon (the HEK3 gene) Briefly, gDNA was added into ddPCR Supermix (Bio-Rad, #1863024), 250 nM probes, and 900 nM primers in a final 20 μL reaction mixture. Probes and primers are listed in Supplementary Table 2. Droplets were generated using a QX200 Manual Droplet Generator (Bio-Rad) (Supplementary Fig. 1b). The thermal cycling protocol was adapted from a previous study. The editing efficiency was calculated by using the ratio of concentrations (copies per μL):

$$\text{editing}\;\text{percentage}=\;\frac{\text{carboxyfluorescein}\;\left(\text{FAM}\right)}{\text{hexachlorofluorescein}\;\left(\text{HEX}\right)}\times 100\%$$

### 4.10 Western blot

Protein extracts from iPSCs were prepared in RIPA buffer (Boston BioProducts, BP-115) supplemented with protease inhibitor cocktail tablets (Roche, 04693159001), followed by sonication (25% power for 5 s). 25 μg of boiled protein were separated on NuPAGE™ Bis-Tris Mini Protein Gels. Proteins were transferred to a 0.45 μm PVDF membrane and probed with an anti-frataxin antibody (Abcam, ab110328, 1:1 000; MilliporeSigma, MABN2313, 1:200). β-Actin (Cell Signaling Technology, 4970, 1:1 000) was used as a loading control. Membranes were subsequently incubated with IRDye 680RD or 800CW secondary antibodies (LI-COR Biosciences, 1:10 000), and protein bands were visualized using the LI-COR Odyssey Imaging System.

### 4.11 Virus-like particles production

Production of VLPs was performed according to a previously established protocol (Geilenkeuser et al., 2025). 12 × 10^6^ HEK293FS cells were seeded in a 15-cm poly-D-lysine-coated dish on Day 0. On Day 1, 3.43 μg of pCMV_ENVLPE^+^, 1.94 μg of pCMV_iPE_P2A_Csy4, 5.44 μg of pU6_FXN_F1_PP7, 5.44 μg of pU6_FXN_R1_PP7, and 2.93 μg of pCMV_VSVG were transfected into HEK293FS cells using Lipofectamine 3000. After 36 h, VLPs were collected using the PEG-it solution.

### 4.12 NGS analysis

NGS primers with forward and reverse adapters are in Supplementary Table 3. Sequencing library preparation has been described previously (Liu et al., 2026).

Raw sequencing data were processed as in previous studies (Chen et al., 2018; Li & Durbin, 2009). We preprocessed the raw sequencing data using fastp (parameters: -m --n_base_limit 0 --qualified_quality_phred 20 -u 0 --length_required 30) as follows: paired-end reads (read1 and read2) were merged into a single read. Reads containing any low-quality base (Q < 20) or unknown base (N) were removed. The filtered reads were then aligned to the amplicon sequence using BWA-MEM with parameters -L 30, 30 -E 1 -O 4, which favor alignments with long deletions over soft clipping for downstream analysis. Unaligned reads, soft-clipped reads, and reads whose alignments did not start and end at the amplicon boundaries were discarded. The remaining reads were examined for single nucleotide variants, deletions, and insertions. We used two methods to calculate the baseline mutation rate for inferring editing fidelity. The first method is using mutation rates at flanking regions as a baseline for each sample, and the second method is using mutation rates in the edited regions in the control sample as a baseline.

### 4.13 Off-target analysis

Off-target NGS primer with forward and reverse adapter are in Supplementary Table 4. Sequencing library preparation has been described previously (Liu et al., 2026). All potential OT sites were identified from Cas-OFFinder, allowing 1-nt DNA or RNA bulge, and mismatch up to 3-nt at 1–12-nt from the PAM. To determine whether attB inserted at OT sites, FASTQ files were processed using CRISPResso2 in NHEJ mode, providing the expected sequence and using default setting.

### 4.14 Statistical analysis

All experiments were done with 1–4 biological replicates. Statistical analyses, including unpaired two-tailed *t*-tests, were conducted using GraphPad Prism 8 (GraphPad Software).

## CRediT authorship contribution statement

**Xuntao Zhou:** Writing – original draft, Validation, Resources, Project administration, Methodology, Formal analysis, Data curation, Conceptualization. **Thomas C. Todeschini:** Data curation. **Tianxiong Yu:** Methodology, Data curation. **Runyuan Wang:** Validation. **Bin Liu:** Resources, Conceptualization. **Annie Collins:** Resources, Methodology. **Whitney Welch:** Data curation. **Aditya Valji Ansodaria:** Resources. **Zhiping Weng:** Resources. **Scot A. Wolfe:** Writing – review & editing, Supervision. **Jonathan K. Watts:** Writing – review & editing, Supervision, Resources, Conceptualization. **Erik J. Sontheimer:** Writing – review & editing, Supervision, Resources, Conceptualization. **Wen Xue:** Writing – review & editing, Supervision, Resources, Project administration, Conceptualization.

## Data availability statement

All data generated or analyzed during this study are included in RAWDATA.

## Declaration of competing interest

The authors declare that they have no known competing financial interests or personal relationships that could have appeared to influence the work reported in this paper.
