# Supplementary material for: TwinPE delivered as RNA or VLP enables precise deletion of GAA repeats in the FXN gene
Source: Cell Insight. 2026 Sep 22;5(6):100368. doi: 10.1016/j.cellin.2026.100368 (PMC13742096; doi:10.1016/j.cellin.2026.100368)
Supplement: Supplementary file 1 [file mmc2.pdf]

Figure S1

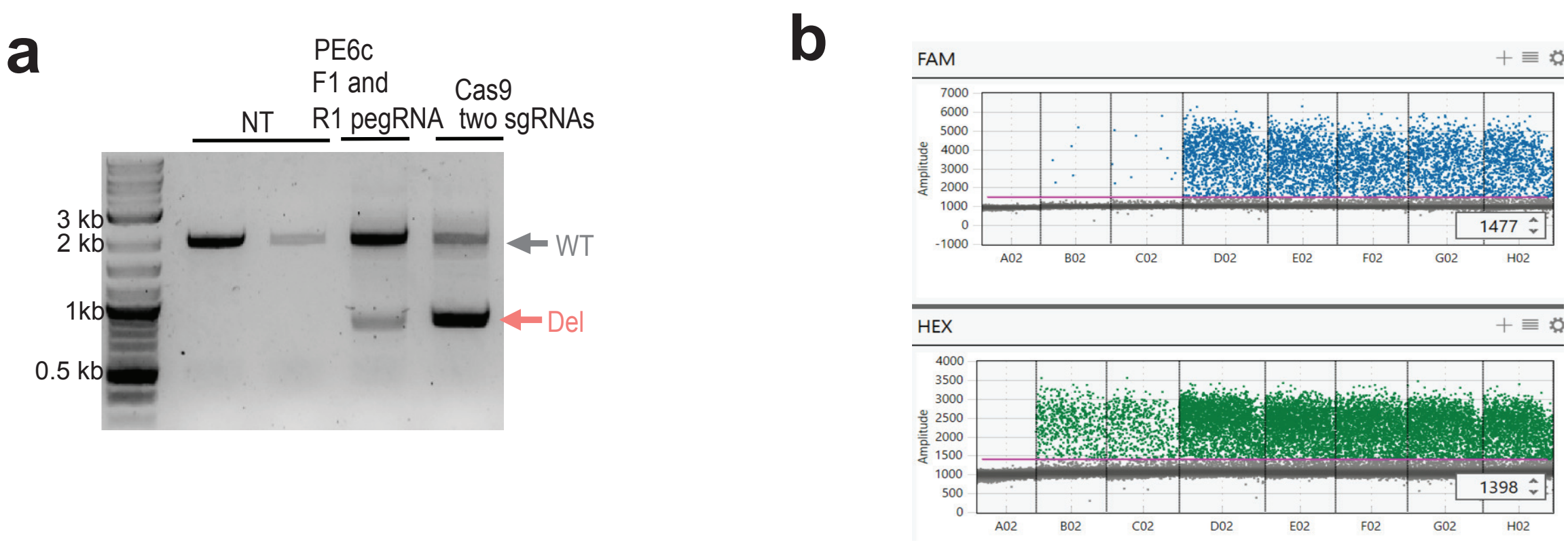

a PCR of analysis of HEK293T cells transfected with PE6c plasmid and epegRNA pairs or Cas9 nickase with sgRNA pairs.
b Representative ddPCR amplitude plots demonstrating specific detection of the edited in *FXN* and *HEK3 (*reference control). A02: water; B02, C02: NT; D02 - H02: edited.

Figure S2

a

**F1**	**TATCTGACCCAGTTACGCCA**	Ch9
F1_OT1	TATCTGgCgCAGgTACGCCA	Ch22, mismatch 3
F1_OT2	TcTC-GAaCCAGgTACGCCA	Ch21, mismatch 3, DNA bulge 1

**R1**	**AATAGCCCTTAACAGCCACC**	Ch9
R1_OT1	gATAcCaCTTAACAGCCACC	Ch9, mismatch 3
R1_OT2	gATAcCaCTTAACAGCCACC	Ch3, mismatch 3
R1_OT3	AAT-GaggTTAACAGCCACC	Ch5, mismatch 3, RNA bugle 1
R1_OT4	A-gcGCtCTTAACAGCCACC	Ch20, mismatch 3, RNA bugle 1
R1_OT5	gATgGCtCCTTAACAGCCACC	Ch7, mismatch 3, DNA bugle 1
R1_OT6	AcT-GCCtgTAACAGCCACC	Ch1, mismatch 3, RNA bugle 1
R1_OT7	cAgAGCCAaTTAACAGCCACC	Ch4, mismatch 3, RNA bugle 1

b

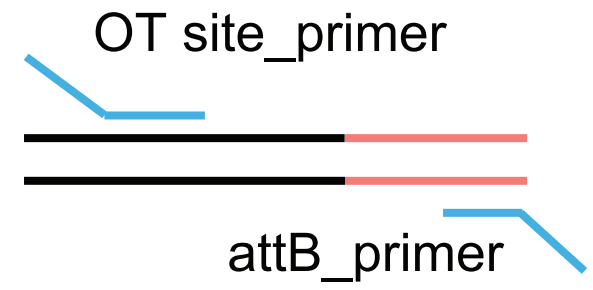

c

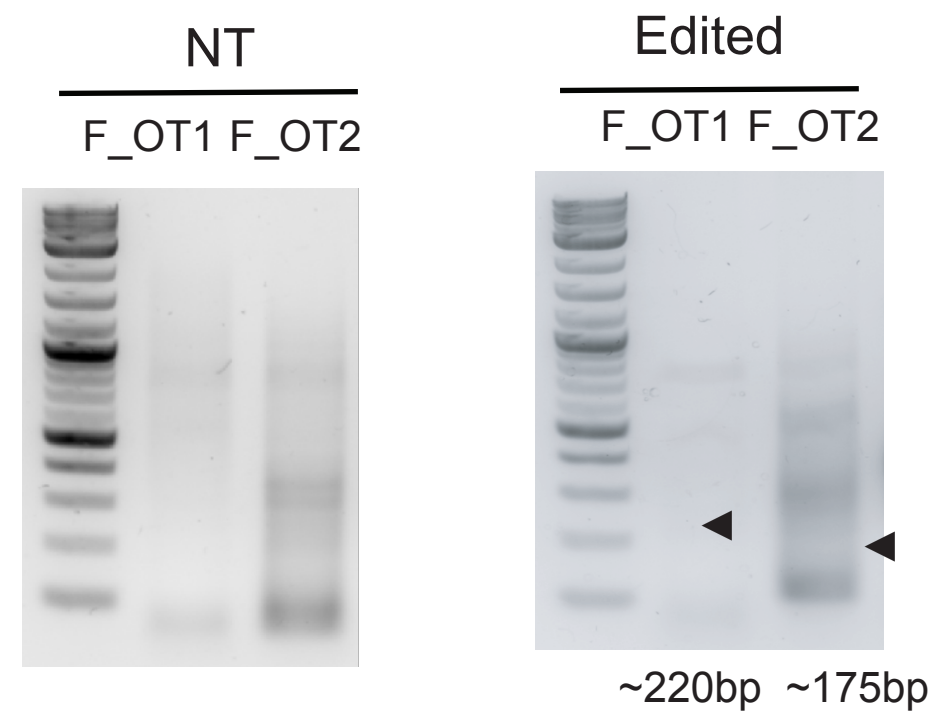

d

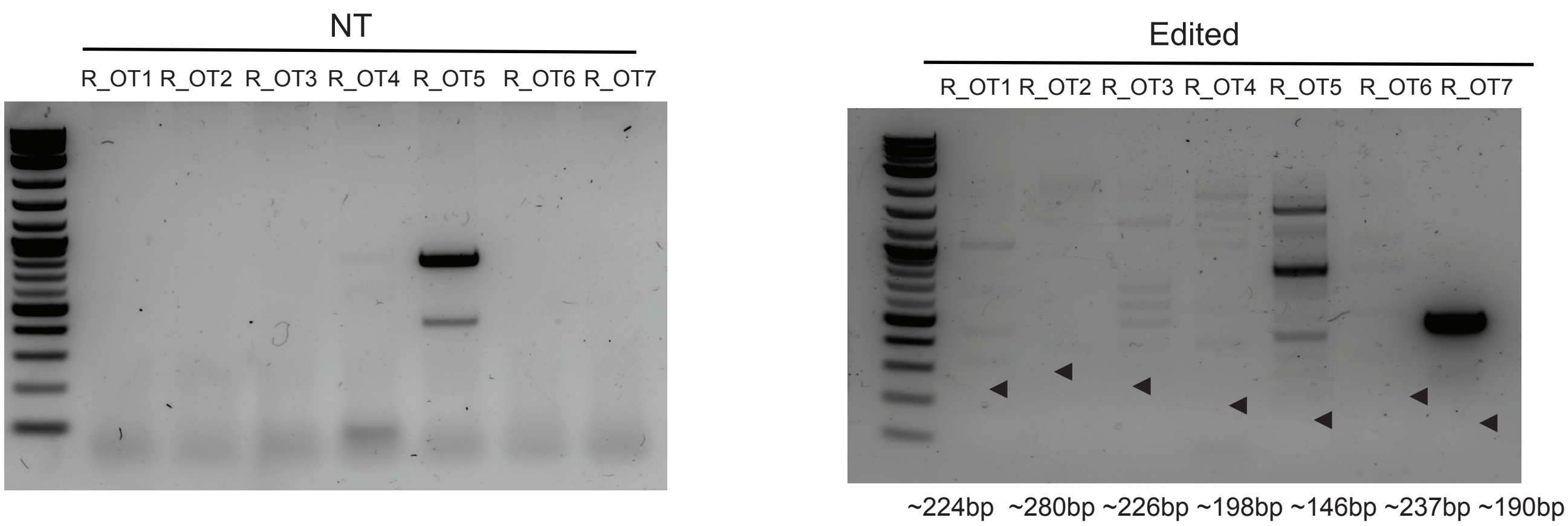

e

	junction NGS
F1_OT1	No alignments
F1_OT2	No alignments
R1_OT1	No alignments
R1_OT2	No alignments
R1_OT3	No alignments
R1_OT4	No alignments
R1_OT5	No alignments
R1_OT6	No alignments
R1_OT7	No alignments

f

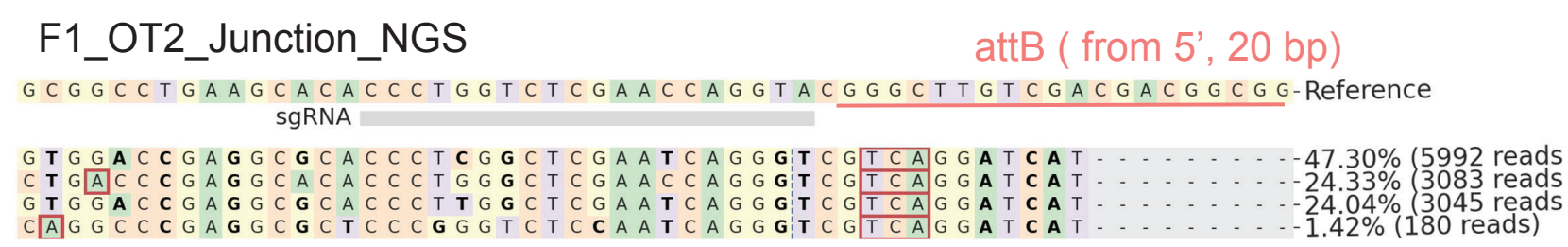

a List of potential off-target sites, including their chromosomal locations, for both the F1 and R1 spacers. Mismatches and DNA/RNA bulges are annotated in red. OT, off target site.
b Schematic of junction PCR primer design.
c-d Junction PCR products from untreated or twinPE-edited gDNA. Arrows indicate the expected band sizes for attB insertion. Expected sizes are annotated below each group.
e-f Deep sequencing analysis of junction PCR products. Aligned reads were obtained after filtering for correct amplicon sequence (containing attB sequence) and a Minimum average read quality (phred33 scale) >30.

Figure S3

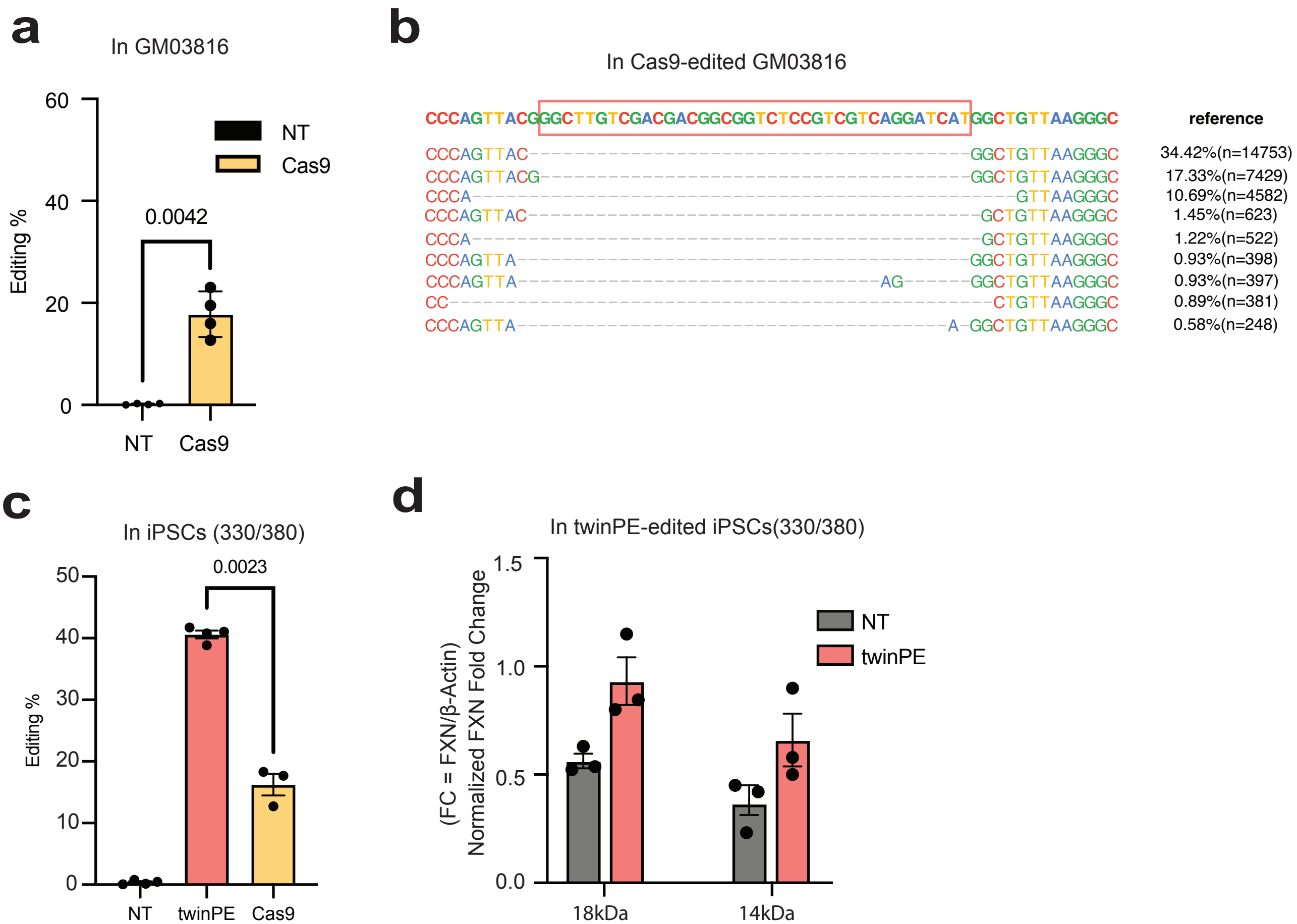

a Editing efficiency at days 3 post-electroporation was measured by ddPCR. Data are presented as mean ± SEM (n=4 biological replicates). NT, non-treated. The amount of Cas9 mRNA was 250 ng, and each of the two sgRNAs was 50 pmol. Statistical significance was determine by an unpaired, two-tailed t-test with Welch's correction.
b Representative deep sequencing of the edited region. Deletions are indicated by dashed lines. No attB insertion(pink box) was detected in the representative reads shown in Cas9-edited GM03816, as expected.
c Editing efficiency at days 3 post-electroporation in iPSCs (330/380) was measured by ddPCR. Data are presented as mean ± SEM (n=4 biological replicates). NT, non-treated. The amount of Cas9 mRNA was 250 ng, and each of the two sgRNAs was 50 pmol.
d Quantification showing increased frataxin expression normalized to β-actin in edited iPSCs (n = 3 biological replicates). NT, non-treated iPSCs; twinPE, edited iPSCs.

Figure S4

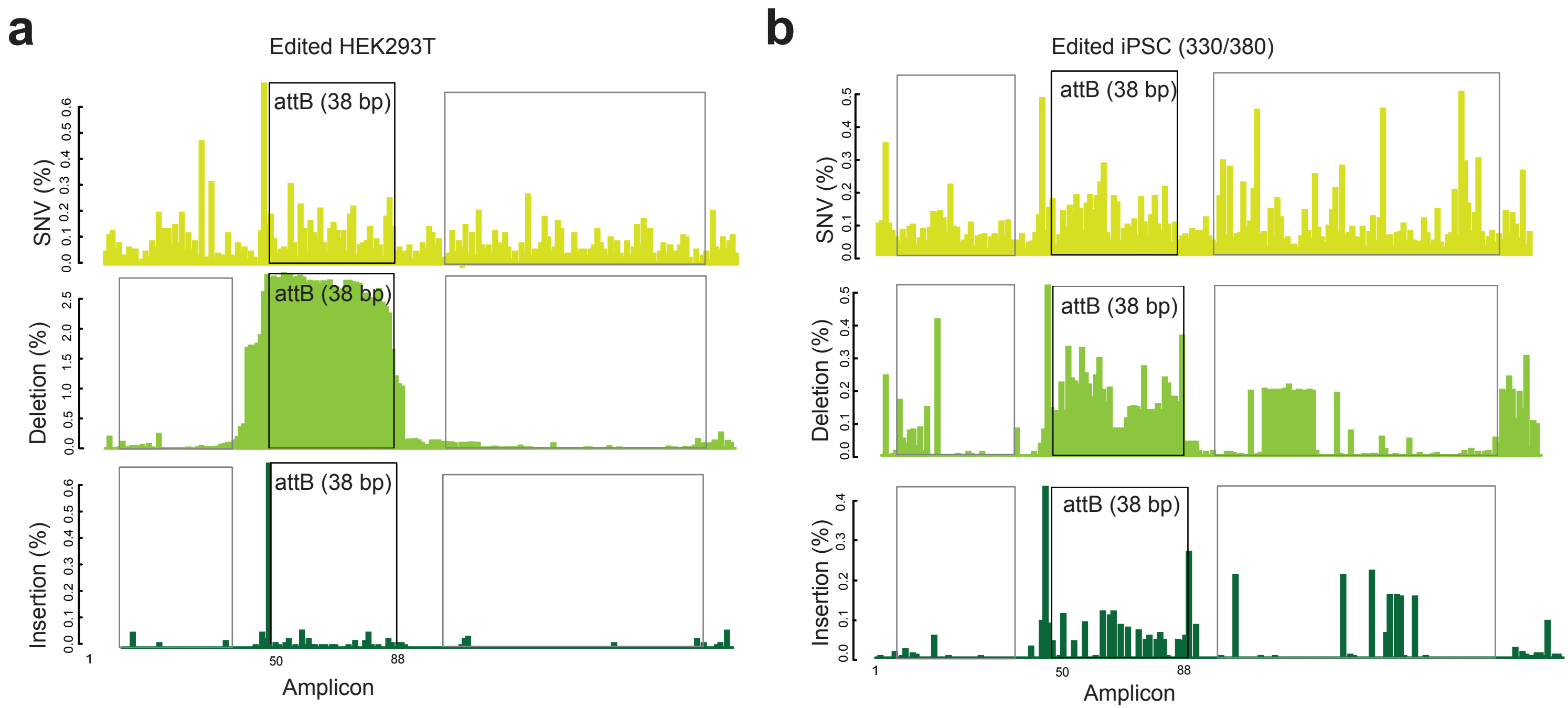

a, b Analysis of editing outcomes by deep sequencing in (**c**) HEK293T cells and (**d**) FRDA iPSC (330/380). The per-base frequency of sequence alterations (y-axis) — including single nucleotide variants (**SNVs**), **deletion**, and **insertion** — is plotted across the 187 bp amplicon (x-axis). Frequencies are shown relative to a baseline calculated from the flanking regions (gray boxes; positions 5–40 and 97–187). The edited region (*attB* ± 5 bp, from position 50 to 88) is highlighted with a black box.

Figure S5

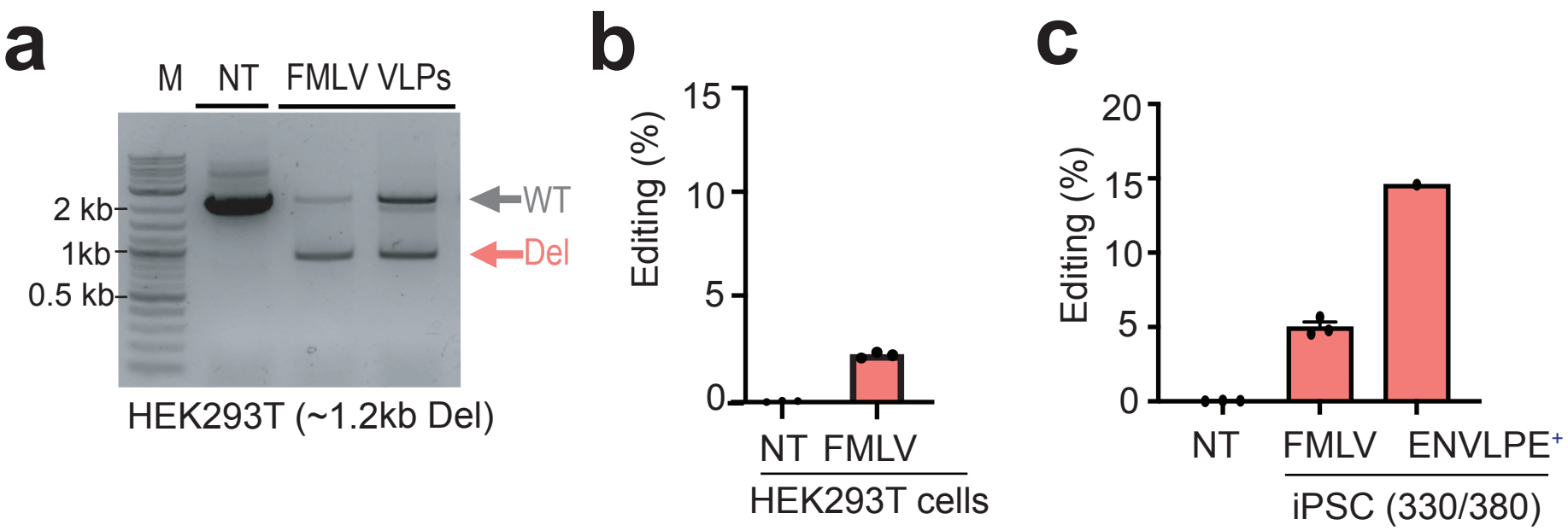

a PCR analysis of HEK293T cells transduced with FMLV VLPs.
b Editing efficiency at days 3 post-transduction was measured by ddPCR. Data are presented as mean ± SEM (n=3 biological replicates).
c In FRDA iPSCs, editing efficiency with different VLPs at D3 post-transduction was measured by ddPCR. Data are present as mean ± SEM (FMLV n=3 biological replicates; ENVLPE^{+} n=1).

Figure S6

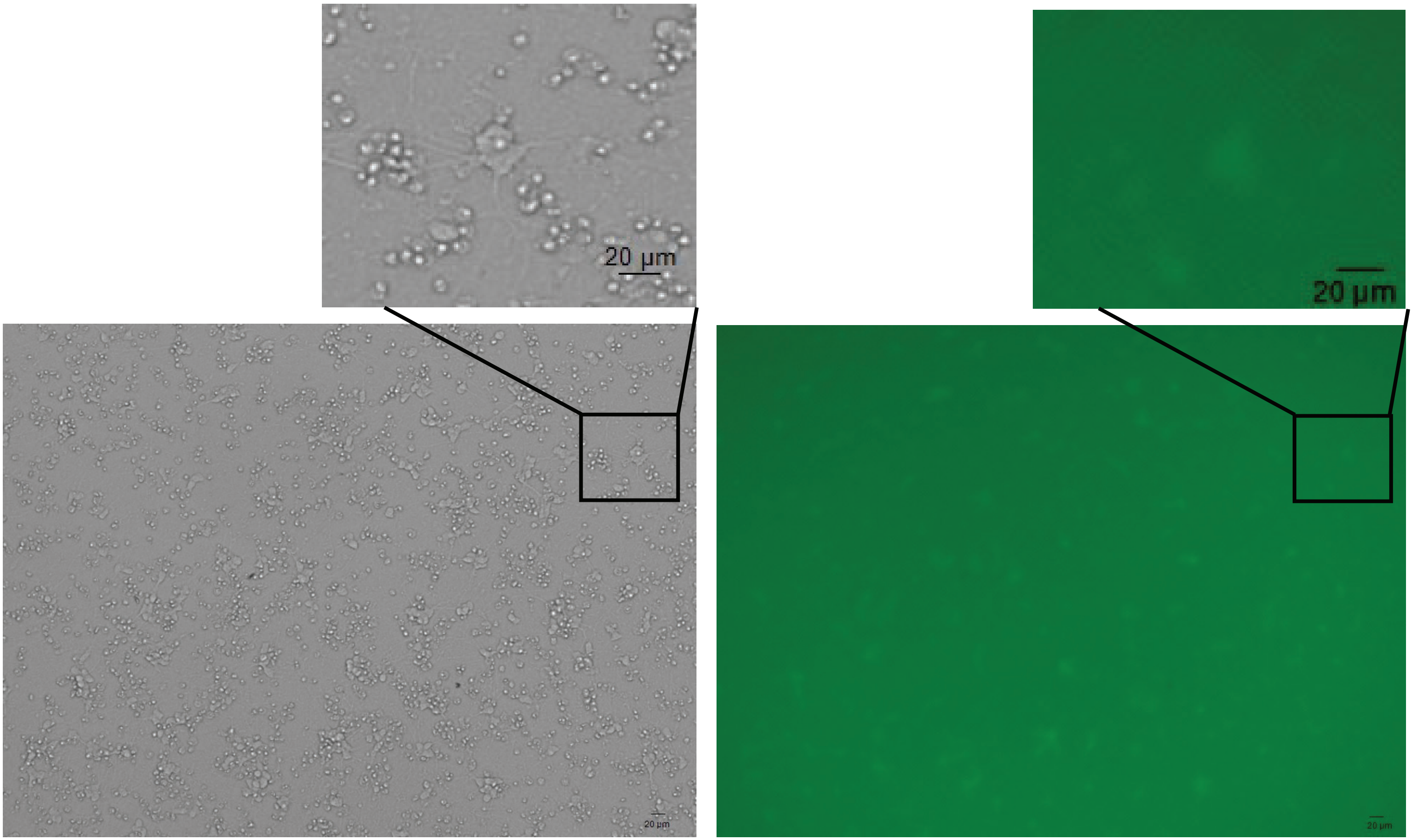

Image FRDA iPSCs (330/380) -derived motor neurons at D25 after ENVLPE⁺ (mNenogreen) transduction.
