# Supplementary material for: TwinPE delivered as RNA or VLP enables precise deletion of GAA repeats in the FXN gene
Source: Cell Insight. 2026 Sep 22;5(6):100368. doi: 10.1016/j.cellin.2026.100368 (PMC13742096; doi:10.1016/j.cellin.2026.100368)
Supplement: Supplementary file 2 [file mmc1.docx]

**Supplementary Information**

**Supplementary Table 1.** epegRNAs

**Supplementary Table 2**. Primers used for PCR, ddPCR and RT-qPCR.

**Supplementary Table 3**. NGS primers.

**Supplementary Table 4**. Off-target analysis primers.

**Supplementary Table 1**. epegRNAs

| **epegRNA** | **spacer** | **RTT** | **PBS** |
| --- | --- | --- | --- |
| F1 epegRNA | gtatctgacccagttacgcca | atgatcctgacgacggagaccgccgtcgtcgacaagcc | cgtaactgggtca |
| R1 epegRNA | gaatagcccttaacagccacc | ggcttgtcgacgacggcggtctccgtcgtcaggatcat | ggctgttaagggc |
| A1 epegRNA | gAACCCAAAGAATGGCTGTG | ATGATCCTGACGACGGAGACCGCCGTCGTCGACAAGCC | AGCCATTCTTTGG |
| A2 epegRNA | GGAAGGGGATCCCTTCAGAG | ATGATCCTGACGACGGAGACCGCCGTCGTCGACAAGCC | TGAAGGGATCCCC |
| A3 epegRNA | GGATGAGGAAGATTCCTCAA | ATGATCCTGACGACGGAGACCGCCGTCGTCGACAAGCC | AGGAATCTTCCTC |
| A4 epegRNA | GTGGTAGAGGGTGTTTCACG | ATGATCCTGACGACGGAGACCGCCGTCGTCGACAAGCC | GAAACACCCTCTA |
| B1 epegRNA | gCTCTGCTGATGGCCAGACG | GGCTTGTCGACGACGGCGGTCTCCGTCGTCAGGATCAT | CTGGCCATCAGCA |
| B2 epegRNA | gGGGGTGGAAGCCCAATACG | GGCTTGTCGACGACGGCGGTCTCCGTCGTCAGGATCAT | ATTGGGCTTCCAC |
| B3 epegRNA | gTAAATGTCTGCTTTCCTAG | GGCTTGTCGACGACGGCGGTCTCCGTCGTCAGGATCAT | GGAAAGCAGACAT |
| F1 with 6xGAA | gtatctgacccagttacgcca | TTCTTCTTCTTCTTCTTC | cgtaactgggtca |
| R1 with 6xGAA | gaatagcccttaacagccacc | GAAGAAGAAGAAGAAGAA | ggctgttaagggc |
| F1 with 6xGAA+20nt 3' | gtatctgacccagttacgcca | gtcaatagcccttaacagccTTCTTCTTCTTCTTCTTC | cgtaactgggtca |
| R1 with 6xGAA+20nt 3' | gaatagcccttaacagccacc | aaatatctgacccagttacgGAAGAAGAAGAAGAAGAA | ggctgttaagggc |

**Supplementary Table 2**. Primers used for PCR.

**2.1** Primers of genomic PCR for deletion and insertion detection

| **Gene locus** | **F (5'-3')** | **R (5'-3')** |
| --- | --- | --- |
| *FXN* | TGGTTCTCCCGGTTGCATTT | AGGGGGAGCTTAGGGTCAAT |

**2.2** Primers and probes for ddPCR

| **Target** | **F (5’-3’)** | **R (5'-3')** | **probe** |
| --- | --- | --- | --- |
| attB | agttcccctttcttcctttggg | gctggtgacagaggttgctaa | CTTGTCGACGACGGCGGTCTCCGTC |
| *HEK3* | GCATGCATTTGTAGGCTTGATG | CAGCCAAACTTGTCAACCAG | CCTGGCCTGGGTCAATCCTTGG |

**2.3** Primers for RT-qPCR

| **Gene locus** | **F (5'-3')** | **R (5'-3')** |
| --- | --- | --- |
| *FXN* (human) | CAGAGGAAACGCTGGACTCT | AGCCAGATTTGCTTGTTTGGC |
| *ACTIN* (human) | TGGACTTCGAGCAAGAGATG | GAAGGAAGGCTGGAAGAGTG |

**Supplementary Table 3**. NGS Primers.

|  | **Adaptor (5’-3’)** | **Primer (5’-3’)** |
| --- | --- | --- |
| Forward | CTACACGACGCTCTTCCGATCT | TCCCCTTTCTTCCTTTGGGG |
| Reverse | AGACGTGTGCTCTTCCGTCT | GCTGGTGACAGAGGTTGCTAA |

**Supplementary Table 4**. Off-target analysis primers.

|  | **Adaptor (5’-3’)** | **Primer (5’-3’)** |
| --- | --- | --- |
| F1_OT1_Forward | CTACACGACGCTCTTCCGATCT | aacaccgggttcatctgcat |
| F1_OT1_Reverse | AGACGTGTGCTCTTCCGTCT | atgatcctgacgacggaga |
| F1_OT2_Forward | CTACACGACGCTCTTCCGATCT | CACGTGCATCACCTTCCCCT |
| F1_OT2_Reverse | AGACGTGTGCTCTTCCGTCT | atgatcctgacgacggaga |
| R1_OT1_Forward | AGACGTGTGCTCTTCCGTCT | ggcttgtcgacgacggcggt |
| R1_OT1_Reverse | CTACACGACGCTCTTCCGATCT | GAAGATGTGCAAATGACCAA |
| R1_OT2_Forward | AGACGTGTGCTCTTCCGTCT | ggcttgtcgacgacggcggt |
| R1_OT2_Reverse | CTACACGACGCTCTTCCGATCT | AACGGAATTGGTTATTAGGG |
| R1_OT3_Forward | AGACGTGTGCTCTTCCGTCT | ggcttgtcgacgacggcggt |
| R1_OT3_Reverse | CTACACGACGCTCTTCCGATCT | TGGCACCTGCTCTGATAGTC |
| R1_OT4_Forward | AGACGTGTGCTCTTCCGTCT | ggcttgtcgacgacggcggt |
| R1_OT4_Reverse | CTACACGACGCTCTTCCGATCT | TCCTAGGTGTCCAGAGAGGC |
| R1_OT5_Forward | AGACGTGTGCTCTTCCGTCT | ggcttgtcgacgacggcggt |
| R1_OT5_Reverse | CTACACGACGCTCTTCCGATCT | GCTCCCCAGAAGGCTGGATA |
| R1_OT6_Forward | AGACGTGTGCTCTTCCGTCT | ggcttgtcgacgacggcggt |
| R1_OT6_Reverse | CTACACGACGCTCTTCCGATCT | CGCCAGCTTTGCCTCTTTTC |
| R1_OT7_Forward | AGACGTGTGCTCTTCCGTCT | ggcttgtcgacgacggcggt |
| R1_OT7_Reverse | CTACACGACGCTCTTCCGATCT | CCCTGTTAAAGTGCGCAATT |
